# Physical Activity and the Association Between Musculoskeletal Complaints and Sleep Quality in Adults and Older Adults From Primary Care: A Cross‐Sectional Study

**DOI:** 10.1002/msc.70280

**Published:** 2026-10-12

**Authors:** Juliana Picchi Kappaum, Rafael Zambelli Pinto, Ana Paula Coelho Figueira Freire, Jamile Sanches Codogno, Bruna Camilo Turi‐Lynch, Rômulo Araújo Fernandes, Henrique Luiz Monteiro, Ítalo Ribeiro Lemes

**Affiliations:** ^1^ Faculdade Israelita de Ciências da Saúde Albert Einstein Einstein Hospital Israelita Sao Paulo Brazil; ^2^ Department of Physical Therapy Universidade Federal de Minas Gerais (UFMG) Belo Horizonte Brazil; ^3^ Physiotherapy Discipline University of Technology Sydney (UTS) Sydney Australia; ^4^ Department of Health Sciences Central Washington University Ellensburg Washington USA; ^5^ Department of Physical Education Sao Paulo State University (UNESP) Presidente Prudente Brazil; ^6^ Department of Exercise Science and Human Performance Lander University Greenwood South Carolina USA; ^7^ Department of Physical Education Sao Paulo State University (UNESP) Bauru Brazil; ^8^ Department of Physical Therapy Sao Paulo State University (UNESP) Presidente Prudente Brazil

**Keywords:** ageing, epidemiology, lifestyle, primary care, sedentary behaviour

## Abstract

**Background:**

Musculoskeletal complaints are the leading cause of disability worldwide and are often associated with poor sleep quality. While evidence suggests that physical activity (PA) positively influences both pain management and sleep quality, it remains unclear whether PA influences the association between musculoskeletal complaints and sleep. This study aimed to investigate the association between musculoskeletal complaints and poor sleep quality, and whether PA modifies this association in adults from primary care.

**Methods:**

This cross‐sectional study analysed data from a Brazilian cohort. Musculoskeletal complaints were defined by self‐reported diagnosis of osteoarthritis or low back pain. Sleep quality was assessed using the Mini‐Sleep Questionnaire. PA was measured using the Baecke questionnaire. Binary logistic regression was used to test the association between musculoskeletal complaints and poor sleep quality.

**Results:**

A total of 557 patients were included (mean age 70.0 ± 8.4 years). Musculoskeletal complaints were associated with poor sleep quality even after adjusting for confounders (OR: 2.36 [95% CI: 1.56 to 3.57]). Stratified analyses showed that this association remained statistically significant among physically inactive participants (OR: 2.47 [95% CI: 1.51 to 4.05]), while the estimate among physically active participants was imprecise and included the null value (OR: 2.10 [95% CI: 0.95 to 4.62]). No statistically significant interaction between musculoskeletal complaints and PA was observed (*p*‐value for interaction = 0.552).

**Conclusions:**

Musculoskeletal complaints are associated with poor sleep quality. Although stratified analyses suggested different effect estimates according to PA, the formal interaction analysis did not support effect modification by PA. These findings should therefore be interpreted cautiously.

## 1 Introduction

Musculoskeletal complaints are one of the leading causes of disability worldwide (Long et al. 2022), with chronic low back pain (LBP) and osteoarthritis (OA) among the most common complaints in adults and older adults (Hartvigsen et al. 2018; Long et al. 2022). Chronic LBP was the primary cause of years lived with disability in most countries, with a global prevalence rate of 15% in adults aged 50 years or older in 2019 (Abbafati et al. 2020). In Brazil, a study showed that 31% of adults aged 55 years or older have chronic LBP (Malta et al. 2022), which is associated with functional dependence and impaired mobility (Dellaroza et al. 2013). In 2019, the prevalence of OA in the global population aged 50 years or over was 24% (Abbafati et al. 2020), while in Brazil, it reached 33% in adults aged over 25 years old and 64% among older adults (Coimbra et al. 2019). Moreover, the incidence of these conditions has increased considerably. For instance, the prevalence of chronic LBP has doubled in recent years in both Brazil (Meucci et al. 2013) and the United States (Freburger et al. 2009). Meanwhile, OA cases have risen by 113% globally between 1990 and 2019 (Long et al. 2022). These data highlight musculoskeletal conditions as a public health problem and reinforce the need for strategies to mitigate their impact.

Previous studies showed an association between sleep quality and musculoskeletal complaints. In this regard, an analysis of patients undergoing treatment for LBP found that 59% of participants experienced sleep problems at some point (Alsaadi et al. 2011). Furthermore, a meta‐analysis revealed that pain intensity (numerical pain rating scale ≥ 7 points) in chronic musculoskeletal conditions is associated with a 2.8 times higher likelihood of insomnia (Bilterys et al. 2021). This relationship seems to be bidirectional, as sleep quality can also affect pain (Runge et al. 2024). Insomnia increases the risk of developing chronic musculoskeletal complaints by 58%, highlighting the role of sleep in pain modulation (Uhlig et al. 2018). A prospective study identified that difficulties in sleep initiation and maintenance, early awakening, and non‐restorative sleep are predictors of chronic musculoskeletal complaints (Aili et al. 2018; Silva et al. 2024).

Sleep quality refers to the ability to initiate and maintain sleep without difficulties and plays a crucial role in physical health (Roth 2007). Inadequate sleep patterns are associated with adverse health outcomes, including all‐cause mortality (Cappuccio et al. 2010) and have a prevalence of 30% among adults (Morin et al. 2006). In this context, regular physical activity (PA) has been identified as a protective factor against insufficient sleep quality (Tian et al. 2024; Tsunoda et al. 2015). A study with older adults demonstrated that those who engaged in moderate low‐intensity PA had 42% lower risk of experiencing insufficient sleep (Tsunoda et al. 2015). Moreover, previous randomized controlled trials showed that structured PA improves sleep quality in women and patients with obstructive sleep apnoea (Arslan and Şevgin 2024; Koçak and Şevgin 2025). These findings highlight PA as a non‐pharmacological strategy for promoting better sleep quality, with benefits for overall health (Bahalayothin et al. 2025).

PA provides numerous health benefits and is considered a key strategy for the prevention and treatment of various chronic diseases (Pedersen and Saltin 2015). Evidence also suggests that PA plays a significant role in pain management, contributing to reduced severity of chronic pain and improved quality of life (Şevgin et al. 2025; Verville et al. 2023). PA is associated with better sleep quality among patients with chronic musculoskeletal complaints (Borges‐Cosic et al. 2019), as well as reduced use of sleep medications (Vanderlinden et al. 2020).

Although PA has a well‐established relationship with both sleep quality and pain, its potential impact on the association between these conditions remains unclear. This lack of evidence is particularly relevant in adults and older adults with multimorbidity, a population characterised by a high prevalence of sleep disturbances (Canever et al. 2024) and musculoskeletal complaints (Coimbra et al. 2019; Dellaroza et al. 2013), a common scenario in primary care. Given the benefits of regular PA in the prevention and management of musculoskeletal complaints and sleep disturbances, understanding its potential role in the association between these conditions may contribute to improving the quality of life by targeting modifiable lifestyle factors. Therefore, this study aims to investigate the association between musculoskeletal complaints and poor sleep quality, and to explore whether this association differs according to PA.

## 2 Methods

This study was approved by the Ethics Committee of (i) the Sao Paulo State University (UNESP), Bauru, Brazil, and (ii) the Einstein Hospital Israelita, Sao Paulo, Brazil. All participants provided written consent to participate in the study. The reporting of this study followed the STrengthening the Reporting of OBservational studies in Epidemiology (STROBE) guidelines.

### 2.1 Study Design, Settings and Participants

The present study is a cross‐sectional analysis of the assessment conducted in 2016 as part of a cohort study, which was started in 2010 (Codogno et al. 2011; Lemes, Sui, Fernandes et al. 2019; Lemes, Sui, Turi‐Lynch et al., 2019; Turi‐Lynch et al. 2019). In this cohort study, waves of assessment were conducted in 2012, 2014, 2016, 2018 and 2020. Participants were recruited from the primary care of the Brazilian National Health System in the city of Bauru, Sao Paulo. Bauru is a medium‐sized city (∼360.000 inhabitants; Human Development Index [HDI] = 0.801) located in the central region of the Sao Paulo State. The primary care of the Brazilian National Health System is composed of Basic Healthcare Units (BHU), which are small healthcare centres that offer low complexity health procedures (e.g., medical consultations, dentistry, nursing, vaccination) to a specific region of the city. All services provided are free of charge to all the population. The 2016 wave was selected because it was the first to include a sleep questionnaire.

At baseline (2010) of the original cohort study, participants were recruited from the biggest BHU of each geographical region of the city (north, south, west, east and central). The sample size was calculated based on the percentage of the population attended exclusively by the Brazilian National Health System (60%), with a standard error of 3.8%, 5% significance (*z* = 1.96), and design effect of 50%, indicating that a total of 960 participants (192 in each BHU) was required. The following inclusion criteria were applied: (i) age ≥ 50 years, (ii) at least 1 year of residence in the area covered by specific BHU, and (iii) at least one medical consultation in the 6 months before baseline measurements. A total of 970 participants were included in the inception of the cohort study.

Because the present study is a secondary cross‐sectional analysis of a longitudinal cohort, the sample size was not originally determined for the current research question. Therefore, a post hoc power analysis was conducted. Based on an odds ratio of 2.8 reported in a previous meta‐analysis (Bilterys et al. 2021), an expected prevalence of poor sleep quality of 45.8% among participants without musculoskeletal complaints, a two‐sided α level of 0.05, 80% statistical power, and an allocation ratio of 2.59 between participants with and without musculoskeletal complaints, the estimated minimum sample size was 156 participants.

### 2.2 Musculoskeletal Complaints

The presence of musculoskeletal complaints was assessed using a specific questionnaire developed by the researchers, which included various chronic conditions (e.g., hypertension, dyslipidemia, type 2 diabetes, cardiovascular diseases, OA, and LBP). The presence of LBP and symptomatic OA was assessed through self‐report, based on whether participants had ever received a medical diagnosis of these conditions (yes/no). For the present study, the occurrence of musculoskeletal complaints was defined by the presence of OA pain or LBP.

### 2.3 Sleep Quality

The Brazilian version of the Mini‐Sleep Questionnaire was used to assess sleep quality (Falavigna et al. 2011). This questionnaire consists of 10 questions and answers are given on a seven‐point scale ranging from 1 (never) to 7 (always). The questionnaire evaluates different aspects of sleep (e.g., sleepiness, insomnia, snoring, difficulty falling asleep, and waking up during the night) and generates a numerical score ranging from 10 to 70 (10–24: good sleep‐wake quality, 25–27: mild sleep‐wake difficulties, 28–30: moderate sleep‐wake difficulties, and > 30: severe sleep‐wake difficulties). In the present study, poor sleep quality was defined as a score ≥ 25. Scores below this threshold indicate good sleep‐wake quality with no evidence of sleep difficulties, while scores of 25 or above reflect the presence of sleep‐wake difficulties, ranging from mild to severe as the score increases (Falavigna et al. 2011).

### 2.4 Physical Activity

PA was assessed through a structured interview using the Baecke questionnaire (Florindo et al. 2004; Florindo and do Rosario Dias de Oliveira Latorre 2003). This questionnaire consists of 16 questions with five response options in a Likert scale format (never, seldom, sometimes, often, very often) and provides a PA score for three domains: occupational (8 questions), sports/exercise (4 questions), and leisure‐time (4 questions). The sum of the scores obtained in each domain comprises the total PA score. Because the Baecke questionnaire provides a continuous, dimensionless score and does not include established cut‐off values to classify physical activity levels, participants with a total score ≥ 75th percentile (the top 25% most active) were classified as “physically active”, following the approach adopted in previous studies using this questionnaire (Codogno et al. 2015; Turi et al. 2015).

### 2.5 Covariates

Sociodemographic (sex, age, socioeconomic status) and health‐related (body mass index [BMI] smoking, hypertension, hypercholesterolaemia and diabetes) variables were used to adjust the multivariable model. Anthropometric measurements were carried out at the BHU by trained researchers. BMI was calculated by dividing weight (kg) by height squared (metres).

### 2.6 Statistical Analysis

Continuous variables were expressed as means and standard deviations (SD), and categorical variables were expressed as frequencies and percentages (%). Binary logistic regression was used to test the association between musculoskeletal complaints and poor sleep quality, generating odds ratios (OR) and 95% confidence intervals (95% CI). A multivariable logistic regression model was further adjusted for potential confounders, including sociodemographic and health‐related variables. To formally assess whether PA modified the association between musculoskeletal complaints and poor sleep quality, an interaction term between musculoskeletal complaints and PA was added to the adjusted logistic regression model. The same procedure was applied in a stratified analysis to investigate the association between musculoskeletal complaints and poor sleep quality in physically active and inactive participants. The Hosmer–Lemeshow goodness‐of‐fit test was used to assess model fit (non‐significant results indicated adequate fit). All analyses were performed using SPSS (version 26.0, SPSS Inc., Chicago, IL, USA), and statistical significance was set at *p* < 0.05.

## 3 Results

A total of 557 participants (57.4% of the initial cohort) were included in the 2016 wave assessment. The reasons for missing participants were incorrect phone numbers or unanswered calls (24.5%; *n* = 238), refusal to continue participating (6.8%; *n* = 66), could not go to the BHU for the interview (2.1%; *n* = 20) and deceased (9.2%; *n* = 89). The mean age was 70.0 ± 8.40 years old (147 men and 410 women), with 381 participants (68.4%) being at least 65 years. Most of the participants (78.1%) were classified as overweight/obese, and the prevalence of hypertension, hypercholesterolaemia and diabetes mellitus was 81.1%, 53.1%, and 38.8%, respectively. Musculoskeletal complaints were present in 72.2% of the sample and 26.9% were classified as physically active (Table 1).

**TABLE 1:** Sociodemographic characteristics of participants.

Variables	Total sample (*n* = 557)
Age (years)	70.0 ± 8.4
Age (categorical)	
< 65 years	176 (31.6)
≥ 65 years	381 (68.4)
Gender (women)	410 (73.6)
BMI (kg/m^2^)	29.5 ± 5.9
BMI (categorical)	
Normal	122 (21.9)
Overweight/obese	435 (78.1)
Waist circumference^a^ (cm)	98.9 ± 13.2
Economic status (score)	25.3 ± 7.6
Current smoker	58 (10.4)
Hypertension	452 (81.1)
Hypercholesterolaemia	296 (53.1)
Diabetes mellitus	216 (38.8)
Musculoskeletal complaints	402 (72.2)
Physically active^b^	150 (26.9)

*Note:* General characteristics of the sample were expressed as mean and standard deviation for continuous variables, and absolute (%) for categorical variables.

Abbreviation: BMI, Body mass index.

^a^ *n* = 526.

^b^ ≥ 75th percentile.

Table 2 shows the association between musculoskeletal complaints and poor sleep quality. In the crude model, musculoskeletal complaints were associated with poor sleep quality (OR: 2.62 [95% CI: 1.79 to 3.83]). After adjusting for sociodemographic and health‐related variables, the association was slightly attenuated (OR: 2.36 [95% CI: 1.56 to 3.57]).

**TABLE 2:** Association between musculoskeletal complaints and poor sleep quality.

Musculoskeletal complaints	Number of participants with poor sleep quality (%)	Model 1	Model 2
		OR (95% CI)	OR (95% CI)
No	71 (45.8)	1.00 (reference)	1.00 (reference)
Yes	277 (68.9)	2.62 (1.79–3.83)	2.36 (1.56–3.57)
*p*‐value^a^	—	—	0.113

*Note:* Model 1: crude. Model 2: adjusted for age, sex, body mass index, socioeconomic status score, smoking, hypertension, hypercholesterolaemia and diabetes.

^a^ Hosmer–Lemeshow test; OR, odds ratio; 95% CI, 95% confidence interval.

The interaction term between musculoskeletal complaints and PA was not statistically significant (OR 1.30 [95% CI: 0.55–3.05), indicating no evidence that PA modified the association between musculoskeletal complaints and poor sleep quality. Thus, stratified analyses were performed as an exploratory analysis. The combination of physical inactivity and musculoskeletal complaints more than doubled the likelihood of poor sleep quality (OR 2.47 [95% CI: 1.51 to 4.05]), even after adjusting for confounders. On the other hand, musculoskeletal complaints were not associated with poor sleep quality among active participants (OR: 2.10 [95% CI: 0.95 to 4.62]) (Figure 1).

**FIGURE 1:**
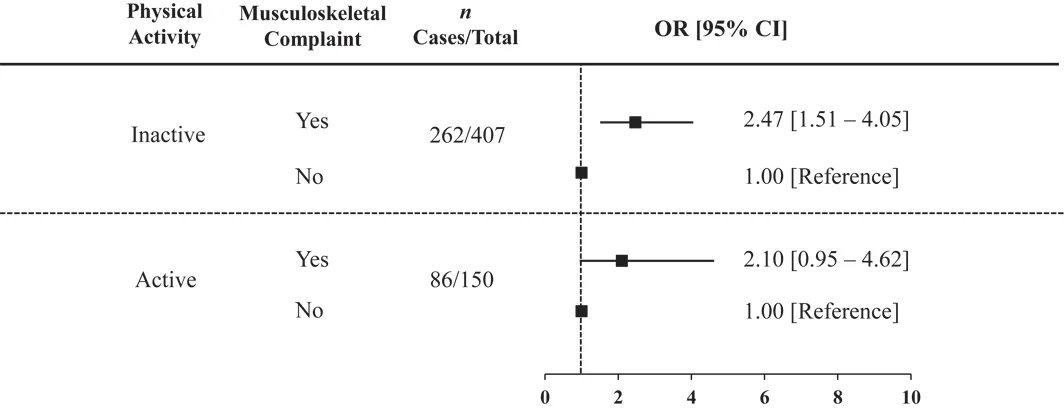
Association between musculoskeletal complaints and poor sleep quality according to PA. Adjusted for age, sex, body mass index, socioeconomic status score, smoking, hypertension, hypercholesterolaemia, and diabetes. 95% CI, 95% confidence interval; OR, odds ratio; PA, physical activity.

## 4 Discussion

This study showed that musculoskeletal complaints are associated with poor sleep quality in adults and older adults from primary care. Individuals with musculoskeletal complaints were 2.4 times more likely to have poor sleep quality compared with those without musculoskeletal complaints, even after accounting for sociodemographic and health‐related variables. Although the stratified analyses suggested different effect estimates according to PA, the formal interaction analysis did not provide statistical evidence that PA modified this association. Therefore, these findings should be interpreted cautiously and considered exploratory.

Our findings corroborate those of a previous systematic review showing that chronic musculoskeletal complaints are associated with sleep problems (Santos et al. 2023). A meta‐analysis revealed that, among patients with chronic musculoskeletal complaints, higher pain intensity is associated with a greater likelihood of insomnia (Bilterys et al. 2021). Furthermore, poor sleep is associated with increased levels of inflammatory markers (Afolalu et al. 2018) that modulate pain perception (Heffner et al. 2011). Inflammatory mediators, such as pro‐inflammatory cytokines, can enhance the sensitivity of sensory neurons, resulting in reduced pain thresholds (Heffner et al. 2011). In this context, our sample was predominantly composed of older adults with overweight/obesity and a high prevalence of chronic cardiometabolic diseases, including hypertension, hypercholesterolaemia, and diabetes mellitus. These conditions are associated with systemic inflammation (Esser et al. 2014; Ferrucci and Fabbri 2018), in which pro‐inflammatory cytokines act on signal transduction across peripheral nerve terminals, the spinal cord, and the vagus nerve (Maier et al. 1998). This neuroimmune interaction may promote central and peripheral sensitisation, thereby increasing pain perception and leading to hyperalgesia, which may influence the observed associations. However, these inflammatory pathways should be interpreted with caution, as such variables were not directly measured in the present study.

Although the formal interaction analysis did not demonstrate that PA modified the association between musculoskeletal complaints and poor sleep quality, the stratified analyses suggested different effect estimates according to PA level. Specifically, the association remained statistically significant only among physically inactive participants, while the estimate among active participants was imprecise and included the null value. Although speculative, one possible explanation for these differences in the point estimates is that physically active individuals tend to adopt healthier lifestyle behaviours that may benefit both musculoskeletal health and sleep quality. A previous meta‐analysis showed that the promotion of PA combined with a healthy lifestyle is effective in reducing musculoskeletal complaints (Herrero et al. 2024). Similarly, studies have shown that positive lifestyle habits, such as stress management and regular PA, contribute to improved sleep health and fatigue (Buğday et al. 2025; Murawski et al. 2018). The reduction of dopamine, serotonin, and GABA levels, along with increased cortisol and pro‐inflammatory cytokine concentrations, are physiological alterations observed in sleep disorders (Levenson et al. 2015), for which regular PA represents a non‐pharmacological treatment strategy (Murray et al. 2017). PA regulates inflammatory markers (Beavers et al. 2010), enhances the synthesis and availability of key neurotransmitters (Lin and Kuo 2013) and contributes to hormonal modulation (De Nys et al. 2022). These mechanisms may partly explain the observed differences in the point estimates between PA groups; however, given the absence of a statistically significant interaction, this interpretation remains speculative. In addition, these inflammatory pathways should be interpreted with caution, as such variables were not directly measured in the present study.

Although no statistical evidence of effect modification was observed, the established benefits of regular PA for musculoskeletal health and sleep quality support its promotion as part of a healthy lifestyle. Engaging in regular PA may help preserve sleep quality even in the presence of musculoskeletal complaints and may also contribute to reducing the use of sleep medications. Considering that musculoskeletal complaints are a leading cause of disability worldwide and place a substantial burden on healthcare services, promoting PA may represent a viable strategy to reduce healthcare demand, especially in primary care settings. In addition, PA has been associated with the regulation of inflammatory markers and neurotransmitters, such as dopamine and serotonin, potentially contributing to overall clinical improvement. Therefore, healthcare professionals may educate patients about the importance of regular PA as part of a comprehensive approach to reduce the impact of musculoskeletal complaints on sleep quality and promote overall quality of life.

### 4.1 Limitations

This study has limitations. First, OA, LBP, and PA were assessed using self‐reported questionnaires, which may have introduced measurement errors and recall bias. Future studies should consider using objective measures of PA (e.g., accelerometers) and standardized clinical assessments of musculoskeletal conditions whenever feasible. Second, the sample was predominantly composed of older adults, which limits the generalisability of the findings to younger populations. Third, defining physically active participants based on the 75th percentile introduces a sample‐dependent threshold that may not reflect clinically meaningful levels of PA, thereby limiting the generalisability of the findings. Fourth, the cross‐sectional design does not allow causal inferences. Further longitudinal studies are needed to better understand the mechanisms underlying these associations. Fifth, participants were classified as having musculoskeletal complaints based on self‐reported physician diagnosis of OA or LBP. Thus, the exposure variable (i.e., musculoskeletal complaints) may reflect the presence of the condition rather than the symptom (i.e., pain) itself. Despite these limitations, the study provides relevant findings on the association between musculoskeletal complaints and poor sleep quality, as well as the role of PA in this association.

## 5 Conclusion

Musculoskeletal complaints are associated with poor sleep quality, regardless of sociodemographic and health‐related confounders. Although the stratified analyses suggested that the association was not statistically significant among physically active participants, the formal interaction analysis did not support effect modification by PA. Therefore, these findings should be interpreted cautiously.

## Author Contributions

**Juliana Picchi Kappaum:** conceptualization, data curation, formal analysis, writing – original draft. **Rafael Zambelli Pinto:** conceptualization, writing – review and editing. **Ana Paula Coelho Figueira Freire:** conceptualization, writing – review and editing. **Jamile Sanches Codogno:** conceptualization, methodology, investigation, data curation, writing – review and editing. **Bruna Camilo Turi‐Lynch:** conceptualization, methodology, investigation, data curation, writing – review and editing. **Rômulo Araújo Fernandes:** conceptualization, methodology, data curation, writing – review and editing. **Henrique Luiz Monteiro:** conceptualization, methodology, writing – review and editing. **Ítalo Ribeiro Lemes:** conceptualization, methodology, investigation, data curation, formal analysis, supervision, writing – review and editing.

## Funding

JPK and IRL were supported by scholarships from São Paulo Research Foundation (FAPESP) grants #2024/10092‐4, #2025/06018‐6, #2015/17777‐3, and #2016/11140‐6, and from the Coordination for Improvement of Higher Education Personnel (CAPES), finance code 001.

## Conflicts of Interest

The authors declare no conflicts of interest.

## Data Availability

The data that support the findings of this study are available from the corresponding author upon reasonable request.
